# Twenty-fourth Annual Report of the Bloomingdale Asylum for the Insane, for the Year 1844

**Published:** 1845-05

**Authors:** 


2. The report before us is the first from the pen of Dr. Earle, since his connexion with the Bloomingdale Asylum, and, as might have been expected, sustains the reputation of its author for industry and talents in the treatment of the insane.

| | Males. | Females. | Total. |
|---|---|---|---|
| Number of patients in the Asylum January 1st, 1844. | 53 | 47 | 100 |
| Admitted during the year, | 57 | 49 | 106 |
| In the Asylum during the year, | 110 | 96 | 206 |
| Discharged during the year, | 56 | 46 | 102 |
| Remaining Dec. 31, 1844, | 54 | 50 | 104 |

Of the patients admitted during the year, there were, of those whose disease was supposed to be of less than one years' duration, and called recent, 69

Of more than one years' duration, and called chronic, 37

Of the patients discharged, there were

| | |
|---|---|
| Recovered, | 50 |
| Improved, | 27 |
| By request, | 12 |
| Died, | 13 |

In relation to the treatment of Insanity, the Doctor says:—

"There are many persons in the community who believe insanity to be purely and exclusively a disease of the mind, and consequently set at nought all medical remedies. Many physicians, also, particularly those of Germany, among whom are Hoffbauer, Heinroth, Reil, Benck, and Langermann, concur in this opinion, so far as regards the seat of the disease, but most of them acknowledge the utility of medicines in effecting a cure. Levret, a French physician, who has adopted the theories of the German school, is the only one, so far as I am informed, who maintains that the disorder may always be cured, if it be curable, by moral treatment alone. On the contrary, it is the opinion of a large majority of the physicians of the present day, that the disease is always bodily, and that the mind appears to be disordered only because the organ, through which its operations are manifested, is not in a condition of health. The latter opinion appears the more rational, as it is more consistent with the general principles of physiology, and with the commonly received ideas of the spiritual nature of man. But how diverse, soever, may be the sentiments of individuals in reference to the seat of the disease, it is now generally conceded that the most effective treatment is that which unites, in judicious proportion, the variety of means included by the two divisions already mentioned."

In regard to the free use of the lancet, as employed by some physicians in the treatment of insanity, the Dr. expresses his opinion as follows :

"It is believed, furthermore, that the vital interests of a portion of the community may, to some extent, be subserved by an expression of our belief in the deleterious consequences of the ordinary mode of commencing the treatment of insanity by repeated and copious bleeding. The abstraction of blood from the veins and arteries of those who are suffering under mental derangement, is but little practised in lunatic asylums, either in Great Britain or the United States. The lancet has probably confirmed and perpetuated more cases of insanity than it has cured."

"There are undoubtedly cases which require the use of the lancet, but they are rare. The indiscriminate employment of this method of depletion is highly injurious and reprehensible."

During the past winter Dr. Earle has adopted a mode of instruction heretofore but little practised in the treatment of the insane. He says,

"A few weeks since I commenced, by way of experiment, a series of lectures on miscellaneous subjects, for the benefit of the patients. My anticipations have been more than realized, in the interest which has thus far been manifested in them, and it is consequently determined to continue them, giving one every week throughout the winter." "The lectures are frequented by from sixty to seventy of the patients, and their conduct is marked by order, decorum and attention. Even the most abstract of all the subjects hitherto discussed, the 'Principles and Analysis of Physical, Intellectual and Moral Beauty,' was far more generally appreciated than might be supposed; and we do not recollect ever to have attended a lecture at which there was a greater degree of quiet and becoming deportment among the audience."

The author's remarks on the subject of "restraints" are judicious and liberal, and his practice corresponding with them, is in accordance with the spirit which would remove everything of the kind from the limbs of the insane, if such a thing could be, without detriment to their comfort.

